# Captivity conditions matter for the gut microbiota of an endangered obligate hibernator

**DOI:** 10.1093/conphys/coae072

**Published:** 2024-10-25

**Authors:** Pauline M L van Leeuwen, Gabriela F Mastromonaco, Nadia Mykytczuk, Albrecht I Schulte-Hostedde

**Affiliations:** Department of Biology, Laurentian University, Sudbury, ON, Canada; Conservation Genetics Laboratory, University of Liège, Liège, Belgium; Reproductive Physiology, Toronto Zoo, Scarborough, ON, Canada; Vale Living with Lakes Centre, Laurentian University, Sudbury, ON, Canada; Department of Biology, Laurentian University, Sudbury, ON, Canada

**Keywords:** Biomarkers, captivity, conservation, hibernation, marmot, microbiota

## Abstract

Conservation breeding programmes include translocations of animals across breeding facilities, both *in* and *ex situ*, and to/from their natural habitat. Newly reintroduced Vancouver Island marmots (VIMs) originating from the captive breeding programme are known to experience high winter mortality once reintroduced. Whilst high winter mortality rates amongst reintroduced VIM populations remain a concern of unknown causes, this health issue could potentially be linked to changes in gut microbiota prior to hibernation. Furthermore, captivity is known to impact the gut microbiota of mammals that could be crucial for hibernation. In this study, we explored the diversity of bacterial communities in the gut of captive marmots during the entire active season, both kept in captivity at *in situ* and *ex situ* facilities, as well as free-ranging marmots during the summer period. Gut microbial diversity was higher in marmots held in *ex situ* facilities, outside of their habitat range, compared to captive marmots held within their habitat range, and in the wild, and differences in composition were also observed. In the entire active season, animals kept in the *ex situ* facility had increased abundance in taxa known to be mucin degraders, sulphate producers and possible cross-feeders, whilst an increase in fibre degraders of *in situ* and free-ranging marmots is potentially linked to diet variation between facilities. These results confirm the interest to transfer animals held at zoos to an *in situ* facility before relocation and expand our understanding of microbiota variation according to hibernation cycles in the context of conservation biology.

## Introduction

Hibernation involves extreme physiological changes in mammals (Bouma *et al.*, 2010; Kurtz *et al.*, 2021), which can affect and are affected by the microbial community (Borbón-García *et al.*, 2017). The Gut Microbial Community (GMC) in particular is known to have several functions including the degradation and fermentation of carbohydrates and proteins to produce short-chain fatty acids (SCFAs), peptides, amino acids and other metabolites and the synthesis of vitamins (McKenney *et al.*, 2018). GMC also have immune functions, such as resisting the colonization of exogenous pathogens and the production of antibiotics (Hooper *et al.*, 2012). Like all organisms, hibernators harbor and interact with their associated microbial communities. Moreover, some hibernators do not feed at all for 5–9 months, making them effective models for examining the effects of extreme dietary changes on the resident GMCs. Previous studies have shown that hibernation leads to a loss of general abundance in bacteria and composition variation in Bacillota (Firmicutes), Bacteroidota (Bacteroidetes) and Verrucomicrobiota in the gut for two ground squirrel species (Carey and Assadi-Porter, 2017; Carey *et al.*, 2013; Stevenson *et al.*, 2014). Whilst those studies show bacteria variation during the annual hibernation cycle, whether those cyclical fluctuations have functional consequences for the host remains to be tested. Additionally, it has been shown that GMCs enable the host to recycle nitrogen into essential nutrients (Regan *et al.*, 2022) that can be particularly important during periods of low food intake, such as hibernation, when animals rely heavily on internal resources for survival.

Specifically, hibernation leads to a loss of general abundance and diversity in bacteria. During torpor, the absolute abundance of the phylum Bacillota is particularly reduced, whereas the phyla Bacteroidota and Verrucomicrobiota are elevated in these studies (Carey *et al.*, 2013; Sommer *et al.*, 2016; Kurtz *et al.*, 2021; Greene *et al.*, 2022), despite not being observed in all hibernating species (Stevenson *et al.*, 2014). Bacillota are generally considered to be fibre degraders and cross-feeders that depend on other microbial taxa and plant material for food degradation (Carey and Assadi-Porter, 2017), and are thus highly dependent on the host and feeding behaviour during the active period. In comparison, the phyla Bacteroidota and Verrucomicrobiota include degraders of host-derived substrates, and most of the SCFAs produced during hibernation result from the metabolism of host-derived secretions (Carey and Assadi-Porter, 2017). Their abundance follows the opposite trend to Bacillota during pre- and post-hibernation periods, although not observed in all hibernating species (Stevenson *et al.*, 2014; Greene *et al.*, 2022).

However, the impacts of the host’s environment are also of interest, and captivity is recognized as a factor in variation of GMCs in wildlife. In many cases, there is a loss of taxonomical diversity and overall abundance of bacteria in captivity compared to wild counterparts (Borbón-García *et al.*, 2017; McKenzie *et al.*, 2017; Rosshart *et al.*, 2017; Chiang *et al.*, 2022), but this pattern is not generalized and specific to the host species (Diaz and Reese, 2021). It has also been observed that patterns of torpor can fluctuate between captive and free-ranging hibernating mammals. Shorter torpor bouts were observed in captive settings, as well as changes in timing of arousals (Geiser *et al.*, 2000). As GMCs variation correlate with hibernation patterns, we postulate that captivity is an important driver of GMCs change in critically endangered hibernating mammals under a conservation programme, such as the Vancouver Island marmot (*Marmota vancouverensis*). Studying GMCs is thus important in conservation biology, especially in captive breeding programmes, because they can influence health, immune function and adaptation to environments, which are key factors for the survival and successful reintroduction of endangered species (Trevelline *et al.*, 2019).

Captive breeding programmes involve frequent translocations of animals across breeding facilities, between *in* and *ex situ* environments, and the natural habitat of the targeted species, as it is the case with the critically endangered Vancouver Island Marmot. The free-ranging population was comprised of <40 individuals in the early 2000s (Lloyd *et al.*, 2019), and the species is now dependent on a captive breeding and reintroduction programme and intensive species management by the Vancouver Island Marmot Recovery Team (VIMRT) (Jackson *et al.*, 2015; Roach, 2017; VIMRT, 2017). Individuals are occasionally removed from the wild for breeding purposes and are first taken to the *in situ* housing facility on Vancouver Island, Canada (Marmot Recovery Center, MRC). VIMs are then moved to *ex situ* facilities such as the Toronto Zoo to breed. Pups born in zoos are transported to another location to breed or released back into the wild after a short stay at the *in situ* facility (VIMRT, 2017). The *ex situ* facility is situated outside of the natural habitat of the marmots and are therefore subject to different environmental conditions. The zoo tries to minimize any differences in protocols by controlling many aspects of the marmot’s living conditions.

One of the main challenges of this captive breeding programme is the higher winter mortality rate of newly reintroduced marmots originating from the captive breeding programme when compared to their wild-reared counterparts (Aaltonen *et al.*, 2009; Jackson *et al.*, 2016; McAdie, 2018). The reason for this elevated mortality rate is unclear, although cardiovascular disease has been identified as the overall leading cause of death during hibernation for captive marmots (44% of the 27 hibernation mortality cases investigated over a 19-year period, McAdie, 2018). In this regard, Aymen *et al.* (2022) observed that captive VIMs exhibit increased lipemia and hypertrophic adipocytes that may lead to the development of metabolic disorders and health concerns over time compared to their wild counterpart. Given the potential roles of the gut microbiota in adipose tissue dysfunction and induced cardiovascular disease in mammals (Yang *et al.*, 2021), we argue that the GMC variation in the active period is dependent of captivity conditions—including diet—in the VIM’s case. Records from the breeding programme indicate that 75% of hibernation deaths in captivity (26.5% of overall mortality in captivity) involved individuals held at *ex situ* facilities, whilst the remaining 25% were held at the *in situ* facility (VIMRT, pers. communication, 2023), indicating potential biological variation between VIMs according to facilities.

Few studies to date have examined variation in GMCs between animals present in wild habitat and *in situ* and *ex situ* facilities within captive breeding programmes (Shiu *et al.*, 2022). Physiological variation has already been observed for VIMs between *in situ* and *ex situ* facilities. Marmots held *in situ* are also known to hibernate longer (on average 24–28 days) than at any other *ex situ* locations (Aymen *et al.*, 2021). Our aim for this study is 2-fold: (i) to study variation of GMCs throughout the active period for marmots at both *ex situ* and *in situ* captive settings and (ii) investigate which is closer to their wild conspecifics. It will allow to identify potential critical links to the poor overwinter survival and metabolism of the newly released captive-reared marmots. Although we expect to find GMC differences between marmots held in two captive facilities, we hypothesize that the variation induced by hibernation over time will follow a similar pattern to that observed in other hibernating species. More specifically we expect a significant reduction in overall microbial abundance and shifts in dominant phyla, such as decreased levels of *Bacillota* and *Bacteroidota*. These changes would support metabolic shifts and nutrient conservation during hibernation, with gut microbiota often recovering to pre-hibernation levels after the hibernation period ends (Carey and Assadi-Porter, 2017; Carey *et al.*, 2013; Stevenson *et al.*, 2014). We argue that holding animals in captivity within their geographical range might offer greater opportunities for microbial transmission from original substrates as well as reduced variation in abiotic conditions such as photoperiodism that might mitigate metabolic alteration, influencing circadian rhythms and hibernation (Ren *et al.*, 2020).

**Table 1:** Information on faecal sample collection according to location and time period regarding hibernation of VIM pairs (Nb pairs) and individuals (Nb alone), with total number of samples (Nb samples). All captive VIM pairs and individuals were sampled on multiple occasions across time, whilst wild VIMs were sampled according to capture/recapture rates

		Number of days after emergence or before entrance in hibernation	VIM location										
			Captive *ex situ* facility (Toronto Zoo, ON)			Captive *in situ* facility (Tony Barret Washington Marmot Recovery Center, BC)			Wild (Vancouver Island, BC)		Total		
			Nb alone	Nb pairs	Nb samples	Nb alone	Nb pairs	Nb samples	Nb alone	Nb samples	Nb alone	Nb pairs	Nb samples
	Period 1	1–7		5	20		6	30					
Post-hibernation	Period 2	8–17		5	15		6	29			3	13	121
	Period 3	19–72		5	19		3	7					
Summer		17/6–11/09	1	8	2/16	4	19	11/53	39	43	44	25	129
	Period 3	60–27		5	9		4	22					
Pre-hibernation	Period 2	25–8	3	6	7/28		5	15				11	114
	Period 1	6–1	3	6	6/21		5	10					
Total			3	9	143	4	24	177	39	43	46	33	364

## Materials and Methods

### Sample collection and information

All methods were approved by the Institutional Animal Care and Use Committee (IACUC) at Laurentian University and by the Toronto Zoo Animal Care and Research Committee (ACRC) under the reference 2018-05-02. A total of 364 faecal samples were collected non-invasively from 79 individuals or pairs of VIMs for this study in 2018 and 2019 from three separate locations: the *ex situ* (Toronto Zoo, *n* = 12), *in situ* facilities (Tony Barret Mount Washington Marmot Recovery Center—MRC, *n* = 28, Table 1) and in their natural habitat on Vancouver Island (*n* = 39). Whilst free-ranging marmots were sampled individually, VIMs in captive housing facilities are paired for mating and pup rearing throughout the year in their enclosures. Since animals sharing enclosures usually defecate in the same area, it was difficult to distinguish which animal the faecal sample originated from, and those samples were therefore treated as belonging to the group of VIMs present in the enclosure. Captive settings for all VIMs are similar to Aymen *et al.* (2021, 2022), without variation across the active period, and detailed in Supplementary Table S1. Briefly, variation between facilities rely on diet and substrate (wood shavings vs straw), as well as the location (British Columbia vs Ontario). For captive VIMs, faecal collection was conducted during morning daily enclosure cleanings for captive animals using gloves if fresh material was found. Faecal samples from free-ranging VIMs were collected opportunistically when present from cage traps on Vancouver Island during daily summer population monitoring using peanut butter as bait. Samples were stored in sealed plastic bags in a cooler in the field until transportation in a −20°C freezer within the day until DNA extraction. A subset of those samples was used for faecal cortisol concentration estimations (*n* = 34, see Supp. Material).

Because trapping free-ranging marmots in remote conditions is challenging and dependent on the presence of faecal matter in traps, only one sample per individual was possible. Sample collection for captive marmots was thus conducted accordingly to sampling dates for wild conspecifics. Two to five separate samples from the same captive VIM pair or individual were collected, ranging from 17 July to 18 September, representing the mid-period of the active season of the VIMs, corresponding to the summer dataset (Table 1).

Sample collection was also conducted longitudinally according to hibernation dates in the two facilities for two periods: pre-hibernation (fall, September–December) and post-hibernation (spring, April–May). First post-hibernation and last pre-hibernation days were designated as the first/last day animals defecated in the enclosure, considered as entrance in hibernation/emergence date. Samples were then opportunistically collected from the first day to 72 days (post-hibernation) and 60 days to last day (pre-hibernation) for each pair (Table 1). Pre- and post-hibernation time periods were divided in sub-section (periods 1, 2, 3) according to the number of days before/after entrance/emergence, period 1 being the closest to hibernation and period 3 the latest. All samples were collected during 2018 and 2019.

Information on individual VIMs including individual sex, age, previous location and date of transfer, birthplace, open air enclosure access and presence of pups in the enclosure was obtained through the VIMRT and Species360 (zoological information management system). Data for paired VIMs were combined for a number of variables: the minimal age of a group, sex (M/F if pair of the two sexes; M if only males present in enclosure) and locations for birthplace for each individual and their sire and dam. For example, if an animal from a pair was born in the wild and another in an *ex situ* facility, the output would be ‘*ex situ* and wild’ and thus considered a category for data analysis.

### DNA extraction and sequencing

DNA extractions from the faecal samples collected were conducted using the Stool DNA Isolation Kit (Norgen Biotek Corp) following the manufacturer’s instructions. Twelve blank extractions were made to control for contamination during the extraction process as well as a mock community sample (ZymoBIOMICS™ Microbial Community DNA Standard) and one PCR negative control. The library preparation and sequencing were performed by Genome Québec Inc., as well as the demultiplexing of the sequence reads. Using their designated library protocol, 2 × 250 bp with 30 000 reads/sample sequencing was completed using broad bacterial primers of the region V4 of the 16S rRNA gene (515F-806R; Caporaso *et al.*, 2012) using an Illumina NextSeq platform (Illumina Biotechnology Co.) on the same sequencing lane in one run.

### Bioinformatics

Sequence reads denoising and amplicon sequence variants (ASVs) picking steps were done with the QIIME2 tool (Bolyen *et al.*, 2019; v. 2019.1), using the DADA2 pipeline (Callahan *et al.*, 2016) with trimming forward reads to a minimum of 200 bp and reverse reads to a minimum of 210 bp based on quality scores. ASVs—or also referred to as bacterial phylotypes—were then screened using a pre-trained Naïve Bayes classifier on weighted Silva v.138 99% OTUs full-length sequences (animal distal gut-trained dataset, Kaehler *et al.*, 2019) for taxonomical association using the q2-feature-classifier implemented in QIIME2 (Bokulich *et al.*, 2018). Sequence alignment and phylogeny building were also conducted in QIIME2. The mock community sample was removed from the dataset for analysis after correct identification of 7/8 bacterial strains to the genus level (8/8 family level; Supplementary Fig. S1).

After data importation in R v.4.0.3 (R Development Core Team, 2009) using the phyloseq package (McMurdie and Holmes, 2013), 38 potential contaminants were identified from the extraction blank from the prevalence-based method using the Decontam package (Davis *et al.*, 2018). Those 38 ASVs were removed from the dataset, as well as extraction blank samples, and sequences assigned to mitochondria and chloroplasts for downstream analysis. Rarefying was conducted at 4544 reads representing the lowest library size (Supplementary Fig. S2) for downstream analysis, apart from differential abundance analysis.

### Statistical analysis

Faith’s PD and Shannon indices in each sample were used as metrics to measure the α-diversity (species richness) of gut bacteria between samples. Differences in index values according to the interaction of current VIM location and time period (post-hibernation, summer and pre-hibernation), place of birth, previous location, parent birthplace, presence of pups, sex, minimal age in pairs and outside access if captive were investigated using restricted maximum likelihood fitting linear mixed-effects models (lmer) with VIM pair/individual and month of sample collection as random effects. Differences in Shannon index according to VIM location and days before/after hibernation according to either the pre- or post-hibernation periods were investigated using restricted maximum likelihood fitting Generalized Additive Models (GAMs). Homogeneity of variance assumptions and normality of the residuals were inspected using visual representations through gam.check (R package mgcv).

**Figure 1:**
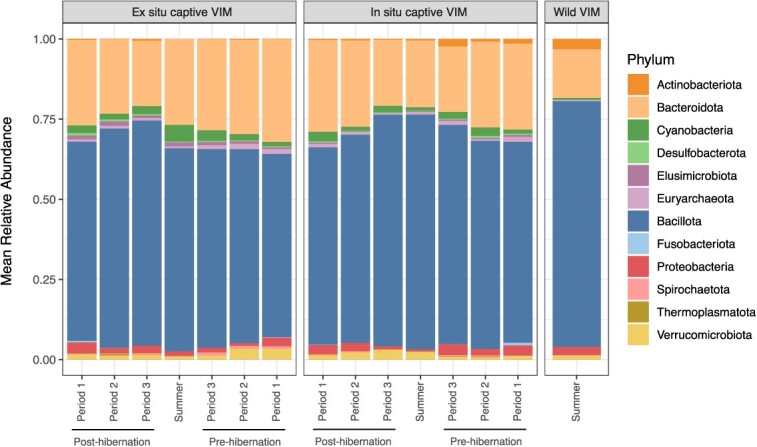
Mean relative abundances of major microbial phyla recovered from *Marmota vancouverensis* (VIM) faecal samples grouped by location in the captive facilities (*ex situ* location and *in situ* location) and free-ranging in the natural habitat (wild) by time period.

Beta diversity was measured through weighted UniFrac distance matrices between samples (Lozupone *et al.*, 2010). Those matrices were used to investigate differences in GMC using PERMANOVA models. Adonis from the *vegan* package were constructed with 9999 permutations and marmot ID as stratification (Oksanen *et al.*, 2019). Differences in samples collected in summer were tested for the following variables: current VIM location, previous location, birthplace, parents’ birthplace, sex minimal age, presence of pups and outside access if captive. For hibernation-related samples, the same variables were used with the addition of time period (pre-hibernation, summer, post-hibernation). Pairwise permutation-based tests of multivariate homogeneity of group dispersions were then conducted to investigate variations between groups with 9999 permutations. Differences in within-group variation were investigated using a betadisper test. Mantel tests were conducted between weighted Unifrac distances and Euclidian distance matrices created based on number of days after emergence or before entrance in hibernation (0 = day of emergence/entrance in captivity).

Finally, we used Analysis of Compositions of Microbiomes with Bias Correction (ANCOM-BC) as method to investigate relative abundances of microbial taxa at the phylum and family levels for time periods (Lin and Peddada, 2020). To ensure accuracy of relative abundances of taxa, we jointly conducted MaAsLin 2 (Microbiome Multivariable Associations with Linear Models, Mallick *et al.*, 2021), using generalized linear and mixed models (negative binomial method) by time periods, with VIM name as random effect, VIM environment as fixed effect and TMM normalization. Significant taxa with corrected *P*-value using the Benjamini–Hochberg method with *P*-value <0.05 in both methods were considered significant.

## Results

### General taxonomy

After reads processing and contaminants filtering, a total read count of 1 654 016 was obtained for gut microbial communities in VIMs. A total number of 31 517 ASVs—or phylotypes—were identified. At the phylum level, Bacillota largely dominated the faecal samples (Fig. 1), representing an average of 66.9% of all samples (SD ±1.2), and Bacteroidota (24% ±9.7) followed by Proteobacteria (2% ±4.1), Verrucomicrobiota (1.9% ±2.1) and Cyanobacteria (1.9% ±2.2). Within the Bacillota phylum, the *Lachnospiraceae* family was the most abundant in captive VIMs (*in situ*: 26% ±8.4; *ex situ*: 23% ±6.1), followed by *Oscillospiraceae* and *Ruminococcaceae*, whilst free-ranging VIMs had the *Clostridia UCG-014* as most abundant family (18.2% ±7.3), then in *Lachnospiraceae*, *Oscillospiraceae* and *Ruminococcaceae*. Amongst the Bacteroidota phylum, the *Muribaculaceae* family was the most prevalent in all marmots (9.9% ±4.6), followed by *Rikenellaceae* and *Bacteroidaceae*.

### Alpha diversity

**Figure 2:**
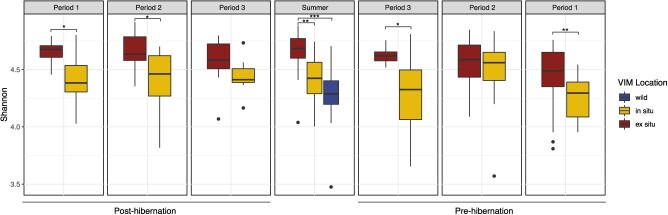
Boxplots of Shannon index variation according to marmot (VIM) location according to temporal effects. *, ** and *** represent significant contrast results of restricted maximum likelihood fitting linear mixed-effects models <0.05, 0.01 and 0.001, respectively.

Gut microbial phylotype alpha diversity did not significantly vary according to faecal cortisol concentration, VIM sex, parent birthplace, presence of pups nor outside access for captive individuals when considering three alpha diversity measures (Supplementary Table S2). The interaction of VIM location and time period had a significant impact on alpha diversity. More specifically, VIMs held in *ex situ* facilities exhibited greater diversity than other locations outside of the mid-pre-hibernation period and post-hibernation period 3 (late summer, Fig. 2, Supplementary Fig. S3). A change in Shannon index was observed according to previous VIM location (F = 5.546, *P*-value <0.01) and birthplace (F = 3.115, *P*-value <0.05), with a greater alpha index when animals were previously located in an *ex situ* facility compared to their respective birthplace, as well as animals born at an *ex situ* compared to *in situ* facility (Supplementary Table S2B). When considering the time periods, GAMs detected a significant reduction of alpha diversity in animals born at the *in situ* facility in the pre- and post-hibernation period compared to *ex situ* VIMs (Supplementary Table S2C). Whilst a significantly greater Faith’s PD index was observable in animals held in zoos from emergence to hibernation up to 60 days, whilst no patterns were identified in *in situ* VIMs (Supplementary Table S2C).

### Beta diversity

**Figure 3:**
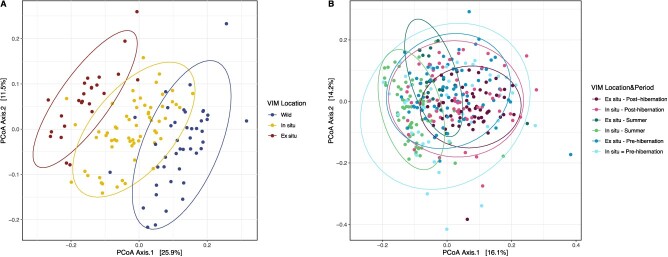
PCoA on weighted Unifrac distances between samples for (A) the summer period according to marmot (VIM) location and (B) pre- and post-hibernation periods according to marmot captive location, with 95% confidence interval ellipses.

In samples collected in summer, dissimilarities in GMC composition measured by weighted Unifrac distances were tested using a PERMANOVA model reflecting dissimilarities based on VIM location, explaining 22.9% of variation between GMCs (Adonis: F = 20.69, R2 = 0.229, *P*-value <0.001; Supplementary Table S3; Fig. 3A). Individual’s birthplace and parents’ birthplace also related to either *ex situ* facilities or the marmot’s natural habitat also exhibited different GMC composition (Adonis: F = 1.79, R2 = 0.049, *P*-value <0.005; F = 1.84, R2 = 0.061, *P*-value <0.005, respectively; Supplementary Table S3). VIMs previously located at an *ex situ* facility also differed in their GMC structure compared to VIMs located at their birthplace or in the wild (Adonis: F = 2.05, R2 = 0.023, *P*-value <0.05).

When considering the time period, VIMs exhibited dissimilar GMC composition according to time period between captive locations, explaining 13.9% of the total variation (Adonis: F = 11.69, R2 = 0.139, *P*-value <0.001; Fig. 3B). VIMs’ birthplace and their parents’ birthplace also significantly explained GMC composition variation (Adonis: F = 5.26, R2 = 0.063, *P*-value <0.001; F = 2.02, R2 = 0.029, *P*-value <0.001, respectively; Supplementary Table S2), as well as previous location (Adonis: F = 2.49, R2 = 0.012, *P*-value <0.001).

Mantel tests revealed positive correlation between number of days before entrance in hibernation and weighted Unifrac distance (r = 0.1545, *P*-value <0.005), with significant positive correlation for *ex situ* captive VIMs (r = 0.1077, *P*-value <0.1) and not *in situ* captive VIMs (r = 0.0722, *P*-value = 0.117). In the same way, with increasing number of days after emergence, the more distant in GMC composition for both *in* and *ex situ* VIMs (r = 0.106, *P*-value <0.1; r = 0.131, *P*-value <0.1, respectively).

**Figure 4:**
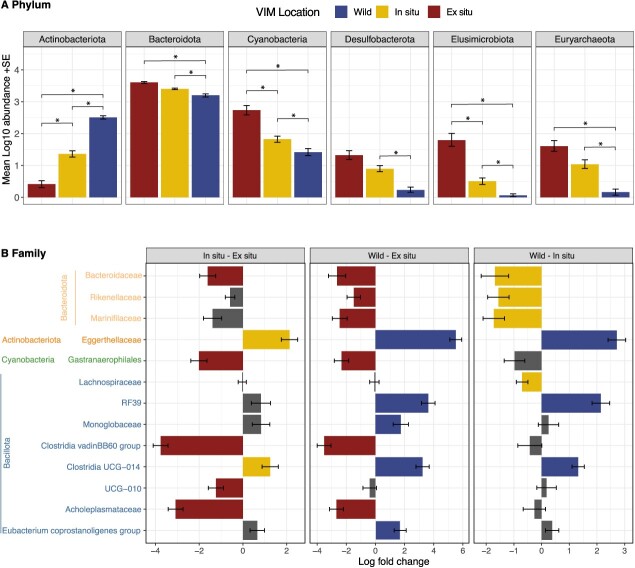
Significant relative abundant (A) phyla (mean log10 abundances by VIM location ± standard error) according to VIM during the summer period. (B) Log fold change variation of microbial families between VIM locations. Colour bars represent in which VIM location the taxa are significantly enriched according to (A), whilst grey bars are non-significant. Only taxa meeting the significance cut-off are represented and colour on taxa name represents its phylum from Fig. 1. ^*^, ^**^ and ^***^ represent significant contrast results of restricted maximum likelihood fitting linear mixed-effects models <0.05, 0.01 and 0.001, respectively.

### Differential abundance analysis

Using ANCOM-BC and Maaslin2, relative abundances of microbial phyla and families within GMCs according to VIM location were assessed during the summer period. At the phylum level, six phyla had significantly greater abundances in *ex situ* VIMs followed by *in situ* VIMs than wild VIMs, including Bacteroidota, Cyanobacteria, Elusimicrobiota and Euryarcheota (Fig. 4A). GMCs from wild VIMs were enriched in Actinobacteriota, whilst captive *in situ* VIMs exhibited greater abundance in Desulfobacterota compared to wild VIMs (Fig. 4A). At the family level, three taxa belonging the Bacteroidota were observed in greater abundance in captive VIMs, as well as Cyanobacteria and three Bacillota families (Fig. 4B). Wild VIMs had their GMC enriched in *Eggerthellaceae* (Actinobacteriota) and five Bacillota families in common with *in situ* VIMs, whilst *in situ-*located VIMs had increased abundance in *Lachnospiraceae*.

**Figure 5:**
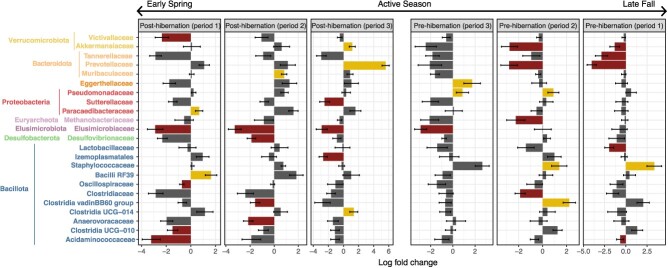
Log fold change variation of microbial families between VIM captive locations according to time period. Colour bars represent in which VIM location the taxa are significantly enriched in (*ex situ*, *in situ*, non-significant taxa). Only taxa meeting the significance cut-off are represented and colour on taxa name represents its phylum from Fig. 1.

When comparing captive locations according to pre- and post-hibernation periods, a greater abundance in the phyla Desulfobacterota and Elusimicrobiota was observed for *ex situ* VIMs during the first period of post-hibernation (logfoldchange = −1.84, SE = 0.4, q-value <0.001; logfoldchange = −2.72, SE = 0.6, q-value <0.001), the second period of hibernation (logfoldchange = −2.19, SE = 0.6, q-value <0.005; logfoldchange = −2.92, SE = 0.7, q-value <0.001) and third period for Elusimicrobiota (logfoldchange = −2.91, SE = 0.7, q-value <0.001). During the pre-hibernation period, *ex situ* VIMs exhibited higher proportions of Elusimicrobiota during the third period (logfoldchange = −2.06, SE = 0.6, q-value <0.05) and Verrucomicrobiota and Euryarcheota during the second period (logfoldchange = −2.44, SE = 0.7, q-value <0.001; logfoldchange = −2.13, SE = 0.6, q-value <0.05). Overall, 16 families exhibited greater abundance strictly in *ex situ* VIMs, 10 during the post-hibernation and 7 in the pre-hibernation periods, compared to nine microbial families for *in situ* VIMs (six taxa enriched in post-hibernation, four in pre-hibernation, Fig. 5). We also observed differential patterns where two taxa (*Prevotellaceae* and *Akkermansiaceae*) were more significantly abundant in *in situ* VIMs in the post-hibernation periods but were enriched in GMC of *ex situ* VIMs during pre-hibernation (Fig. 5).

## Discussion

### GMC variation in summer could be explained by diet differences between captive facilities

Examination of GMC variation, including alpha and beta diversity and differential abundance analyses, yields relatively similar patterns in VIMs held in different environments during the summer. Greater diversity and compositional changes were observed in the GMCs of VIMs held in *ex situ* facilities (zoos) compared to those in the *in situ* facility and the wild. Increased microbial richness in captive mammals is uncommon compared to semi-wild and wild counterparts (Borbón-García *et al.*, 2017; McKenzie *et al.*, 2017; Tang *et al.*, 2020). Although both captive groups had access to outdoor enclosures, the gut microbiota of *in situ* VIMs may be colonized by microbes transmitted horizontally from substrates shared with free-ranging VIMs (Perofsky *et al.*, 2019). Geographical location differences, such as photoperiod variation between Vancouver Island and the Toronto Zoo, may also play a role. Vancouver Island experiences more seasonal daylight variation, with up to 16.5 h in summer and 8.5 h in winter, compared to Toronto’s 15.5 h in summer and 9 h in winter. Other studies have shown GMC changes in response to day length in hibernating Siberian hamsters (Bailey *et al.*, 2010; Ren *et al.*, 2020). Further investigation in controlled environments could explore the impact of photoperiod on VIM GMCs, especially since hibernation duration differs between captive facilities (Aymen *et al.*, 2021).

However, diet variation is a known major driver of GMCs in herbivores and rodents, particularly when comparing captive and free-ranging animals (Frankel *et al.*, 2019; Van Leeuwen *et al.*, 2020). Diet differences between *in situ* and *ex situ* VIMs may explain the observed GMC variation, depending on what marmots are fed during the active season (Dallas and Warne, 2022). At the *in situ* facility, the pellet diet (16%) is supplemented with natural vegetation, including abundant *Lupinus* species, collected from the VIM’s natural habitat (McAddie, pers. communication). Providing food from the species’ natural habitat may expose marmots to microbes that specialize in their diet, leading to GMCs more reflective of free-ranging marmots (Martinez-Mota *et al.*, 2019; Van Leeuwen *et al.*, 2020).

At the Toronto Zoo, the pellet diet is supplemented with raw vegetables (lettuce, kale, broccoli and cauliflower) and occasionally with *Populus* and *Malus* browse (Wensvoort, pers. communication, 2021). Dietary variation may promote different gut metabolic pathways, increasing ecological niches within the marmot’s gut and leading to higher microbial diversity and greater inter-individual variation. The greater variety of raw food items at the *ex situ* facility could explain the greater microbial richness, although feeding trials would confirm this hypothesis. Several taxa, more abundant in *ex situ* VIMs, are known for producing butyrate through carbohydrate fermentation in herbivorous mammals, including *Gastranaerophilales* (Di Rienzi *et al.*, 2013) and *Rikenellaceae* (Chung *et al.*, 2020). This could be explained by the higher proportion of crude fibres in the *in situ* diet compared to the *ex situ* diet, favouring microbes that are not fibre degraders.

GMC variation also depended on the VIMs’ birthplace and that of their parents, highlighting the importance of horizontal transmission and early-life exposure to microbes from the natural habitat (Bokulich *et al.*, 2016; Sonnenburg *et al.*, 2016). The variation in GMCs based on previous locations suggests that *ex situ* VIMs could carry legacy effects from their former environments. Longitudinal studies involving diet manipulation or translocation from zoos to *in situ* facilities before reintroduction are needed to understand how these changes occur. Further investigation between zoos where VIMs are held in similar conditions could also be undertaken.

### Relative abundances in pre- and post-hibernation periods potentially involving key hydrogen and sulphur utilization pathways

Alpha diversity analysis showed that microbial richness declined as the VIMs neared hibernation. Bacterial diversity typically decreases during hibernation (Carey and Assadi-Porter, 2017) as GMCs adapt to the changes in the gut’s physical and metabolic environment, resulting from the reduction of metabolizable substrates. However, different trends were observed in the pre-hibernation period depending on the VIM’s location. As documented by Aymen *et al.* (2021), wild-born and *in situ* VIMs are known to hibernate longer (24–28 days) than *ex situ* VIMs, with the closest similarities between captive and natural hibernation patterns observed *in situ* (Bryant and McAdie, 2003). This is consistent with our summer findings, where *in situ* VIMs had GMCs more similar to free-ranging VIMs than their *ex situ* counterparts. Thus, *in situ* VIMs may experience a loss in microbial diversity that mirrors the natural pre-hibernation period, whilst different dynamics are seen in *ex situ* VIMs, as they exhibit overall greatest microbial richness in this period. These results provide first evidence that captive settings can impact GMCs during the active season of the VIMs. However, the ultimate causes of the increase in microbial diversity remain unclear but could be linked to diverse and differential food intake for *ex situ* VIMs.

Beta diversity analysis also revealed significant differences in GMC composition depending on VIM location and season. These results, consistent with summer findings, may be linked to differences in diet between facilities during the active season, as well as early microbial colonization based on birth location (Spor *et al.*, 2011; Metcalf *et al.*, 2017). Due to the limitations of 16S rRNA short amplicon metabarcoding, it remains unclear whether the observed GMC changes lead to significant functional differences in microbial activity. However, some taxa were differentially abundant between VIM locations in both pre- and post-hibernation periods.

We observed a significant increase in relative abundances of *Elusimicrobiota, Euryarchaeota* and *Desulfobacterota* in captive VIMs, particularly in *ex situ* individuals during both pre- and post-hibernation periods. *Elusimicrobiota*, which are gut-associated, rely exclusively on fermentation for energy and are capable of producing acetate, lactate, hydrogen and sulphur as byproducts of glycogen metabolism (Méheust *et al.*, 2020). The sulphate potentially produced by *Akkermansiaceae* (Verrucomicrobiota) from mucin degradation (Van Herreweghen *et al.*, 2018) could stimulate sulphate reducers such as the archaeal methanogens and *Desulfobacterota* (Moissl-Eichinger *et al.*, 2018; Rath *et al.*, 2018) potentially explaining common enrichment in *ex situ* VIMs. However, excess concentrations of the resulting H_2_S are associated with inflammation of the gut epithelium in humans (Rath *et al.*, 2018). Moreover, an excess in mucin degradation has been linked to loss of host mucus homeostasis and metabolic disorders in mice and humans (reviewed in Tailford *et al.*, 2015), that could potentially be linked with increased lipemia and hypertrophic adipocytes in captive VIMs (Aymen *et al.*, 2022). Because *ex situ* VIMs experience shorter hibernation periods (Aymen *et al.*, 2021) and captive-born VIMs exhibit greater overwinter mortality once relocated (McAdie, 2018), it is possible that microbial dynamics during captive hibernation are different and potentially detrimental to the host than during natural hibernation due to physical and metabolic variation according to environmental factors between captivity and the natural habitat (Sonoyama *et al.*, 2009; Kohl *et al.*, 2014).

## Conclusion

Overall, this study provides the first report of diversity and composition of GMCs in VIMs during the active season. Given the pattern in relative abundances between pre-, summer and post-hibernation periods, *ex situ* VIMs exhibited distinct microbial communities, potentially reflecting functional diversity. Our results show that the current and past locations of VIMs may contribute to differences in the GMCs of captive versus wild VIMs. These variations could be linked to differences in diet at different locations and the types of bacteria available for early gut colonization. Due to the low overwinter survival of captive VIMs released into the wild (Jackson *et al.*, 2016), the gut microbiota that develops in captivity may negatively affect their survival in the wild (Aymen *et al.*, 2022). In light of our results, reintroduction into the natural habitat may be beneficial for VIM health by promoting development of a GMC that more closely mimics that of wild VIMs. Allowing VIMs to undergo at least one hibernation at the *in situ* facility before reintroduction and providing a diet that more closely resembles VIM diets in the wild at the *ex situ* facilities could reduce GMC variation and minimize adaptation to captivity. The implementation of management measures such as the stepping-stone approach for the VIM reintroduction increased captive-born VIM survival (Lloyd *et al.*, 2019). However, further research on translocation strategies between captive facilities could also benefit the VIM captive breeding programme. Microbiome analyses could be a useful tool for government policymakers (Trevelline *et al.*, 2019), improving the current management plans of emblematic and threatened wild species such as the VIM, whose populations have been reduced and for which little is known about the current diet and potential implications for hibernation success. Importantly, these results broaden our understanding of the effects of hibernation on gut microbiota of mammal hibernators under conservation breeding programmes.

## Supplementary Material

Web_Material_coae072

## Data Availability

VIM2022-code.R is available in the figshare repository, as well as metadata, taxonomy, sequences and biom file: Van Leeuwen, Pauline (2023): Gut microbiota of Vancouver Island marmots. Figshare. Dataset. https://doi.org/10.6084/m9.figshare.21967838.v1.
