# Supplementary material for: Captivity conditions matter for the gut microbiota of an endangered obligate hibernator
Source: Conserv Physiol. 2024 Oct 25;12(1):coae072. doi: 10.1093/conphys/coae072 (PMC11503477; doi:10.1093/conphys/coae072)

Supplementary material description from: Captivity conditions matter for the gut microbiota of an endangered obligate hibernator

Authors and affiliations: Pauline van Leeuwen[1,2], Gabriela F. Mastromonaco[3] , Nadia Mykytczuk[4], Albrecht I. Schulte-Hostedde[1]

[1] Department of Biology, Laurentian University, Sudbury, ON, Canada;

[2] Conservation Genetics Laboratory, University of Liège, Liège, Belgium;

[3] Reproductive Physiology, Toronto Zoo, Scarborough, ON, Canada;

[4] Vale Living with Lakes Centre, Laurentian University, Sudbury, ON, Canada

Figure S1. (A) Expected distribution and identification of mock community, extracted from ZymoBIOTICS microbiome standards (Zymo Research) and (B) observed distribution and identification in this study.

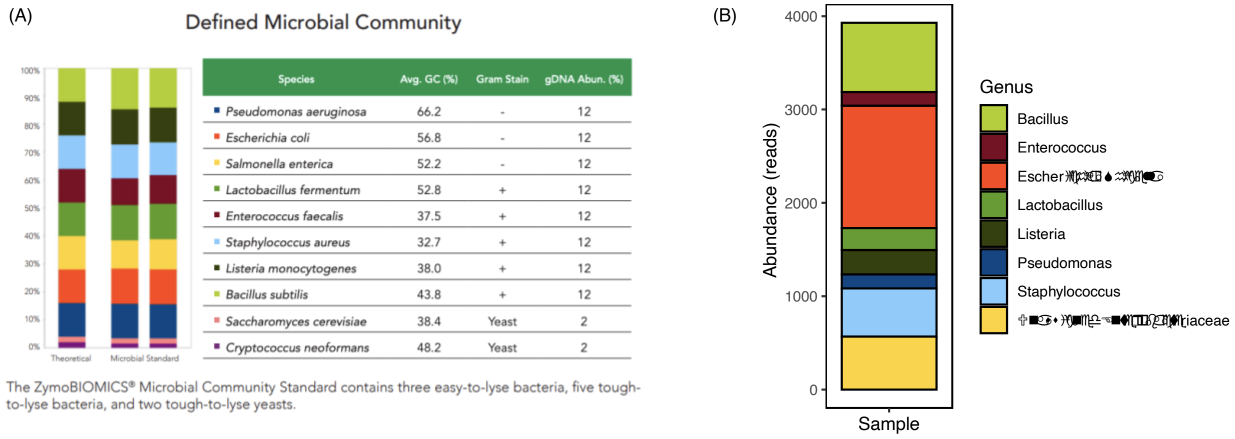

Figure S2. Rarefaction curve of all samples after mock and extraction blanks removal.

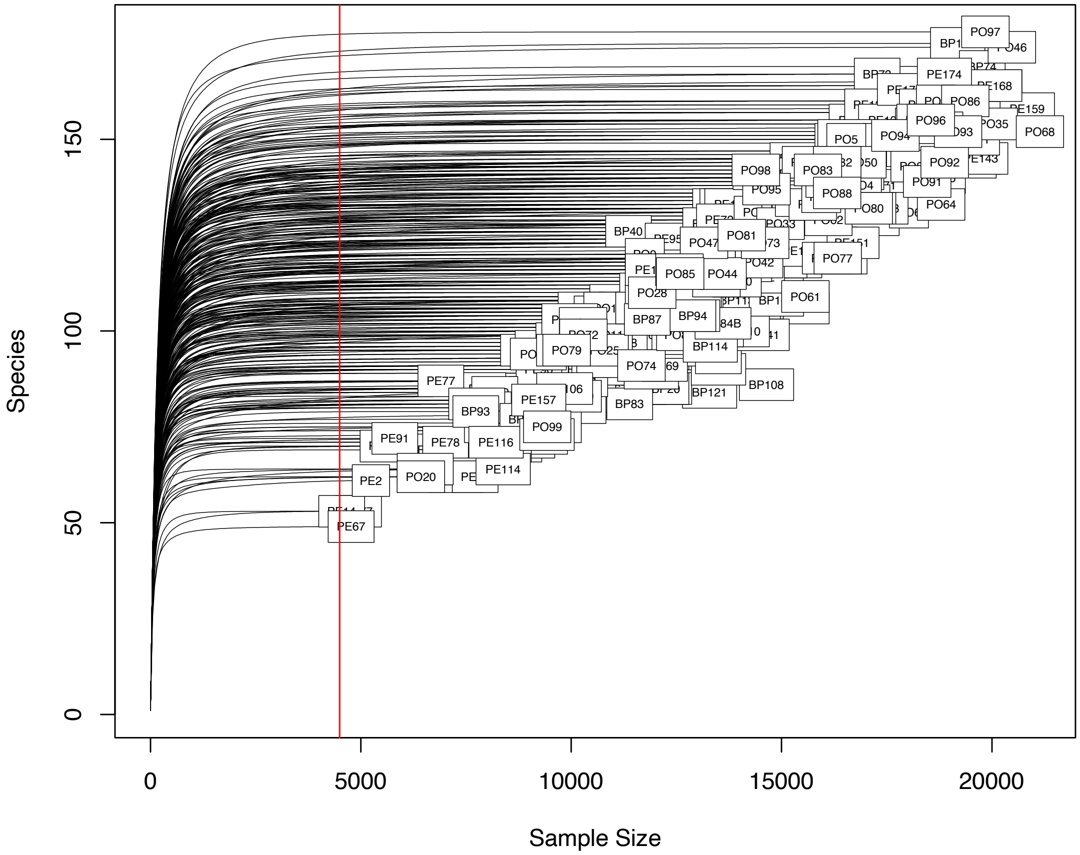

Figure S3. Boxplots of (A) Faith's PD index and (B) Observed ASVs variation according to marmot location according to temporal effects. *, **, *** represent significant contrast results of restricted maximum likelihood fitting linear mixed-effects models below 0.05, 0.01 and 0.001 respectively.

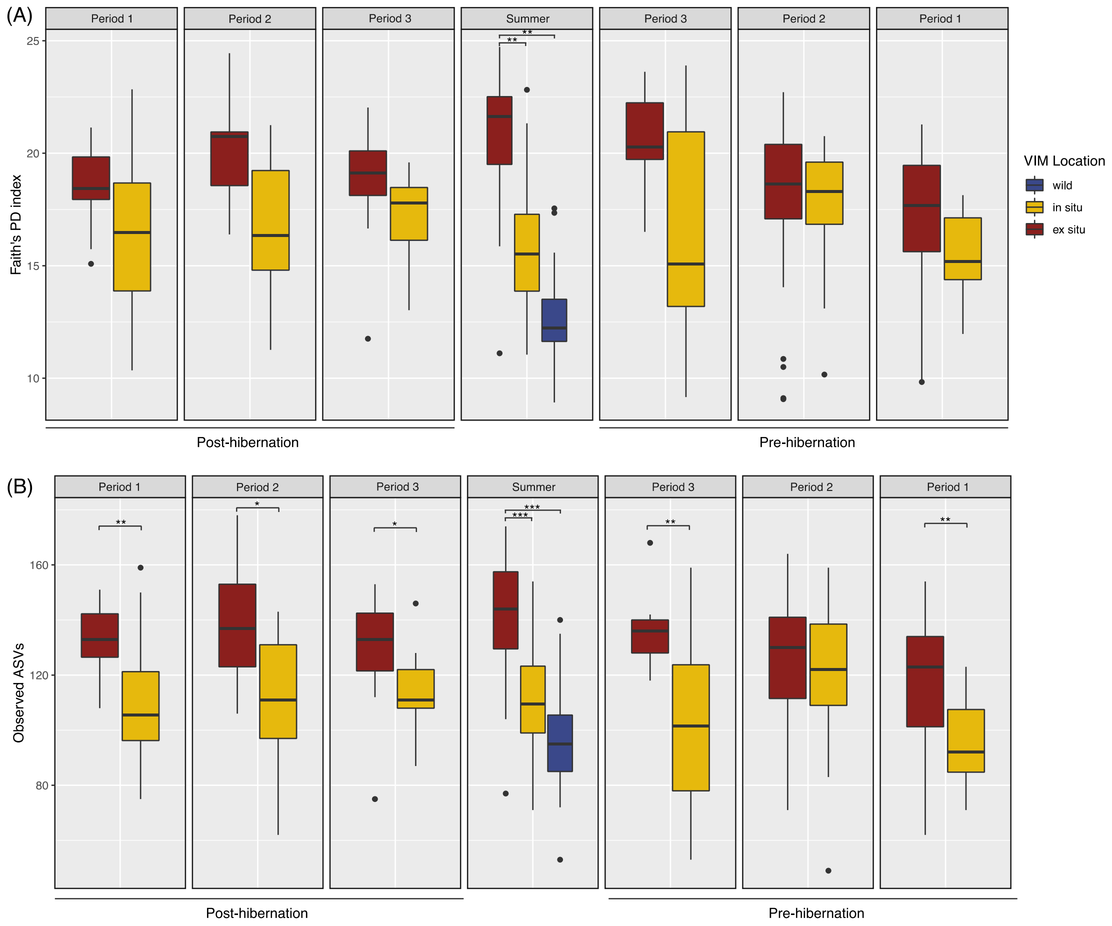

Table S1. Detailed comparison of captivity conditions between the *ex situ* and *in situ* facilities.

	In situ facility	*Ex situ* facility
Outside access	During active season	During active season
Animals by enclosure	Adult pair (± pups)	Adult pair (± pups)
Outside enclosure	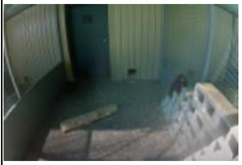	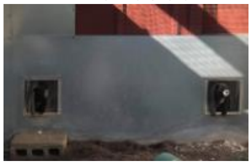
Inside enclosure	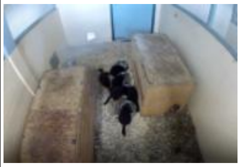	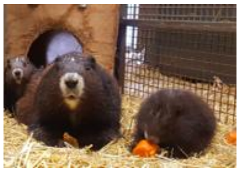
Public display	No	No
Controlled access	Yes	Yes
Protective equipment for keepers	Yes	Yes
Quarantine for marmots new to site	30 days	30 days
Substrates	Wood shavings	Straw
Pellet composition	Crude protein 16%<br>Crude fat 3%<br>Crude fibre 24%<br>Sodium 0.4%<br>Calcium 1%<br>Phosphorous 0.5%<br>Vitamines A,D,E and Selenium	Crude protein 15%<br>Crude fat 3%<br>Crude fibre 14%<br>Sodium 0.2%<br>Calcium 1%<br>Phosphorous 0.55%<br>Vitamines A,D,E and Selenium
Additional raw foods	Lupinus sp (from natural habitat)	Populus sp<br>Apple tree<br>Lettuce<br>Cauliflower<br>Kale<br>Broccoli<br>Carrots
Food access during hibernation	No	No
Access to water during hibernatio	No	At the end of hibernation
Temperature	Controlled and modified for hibernation	Controlled and modified for hibernation
Temperature during hibernation	5-8°C	5-6°C
Light	Natural photoperiod when outside access	Natural photoperiod when outside access
Humidity	80% on average in nest boxes	80% on average in nest boxes
Prophylaxis	Common to breeding program	Common to breeding program
Location	Vancouver Island (BC, Canada)	Toronto (ON, Canada)

Table S2. (A) Results of linear models on both alpha diversity indices, bold values are significant and (B) contrast among least-squares means for significant predictors, only significant values are shown. (C) General Additive Models results of restricted maximum likelihood fitting for hibernation periods investigated according to predictor variables of interest for three alpha diversity indices. De: Deviance explained by each model.

(A) Linear models

Predictors	Shannon index		Faith's PD		Observed ASVs	
	F value	Pr(>F)	F value	Pr(>F)	F value	Pr(>F)
Birthplace	**3.1150**	**0.0440201**	2.4966	0.0510323	**3.1002**	**0.0314006**
Previous location	**5.5458**	**0.0088723**	**3.2640**	**0.0463824**	**5.5602**	**0.0074465**
Sex	1.5510	0.2254260	0.4559	0.6357749	1.6960	0.1936723
Parents' birthplace	1.5329	0.2115851	0.3434	0.9100500	1.4035	0.2438069
Pups presence	0.0414	0.8391830	0.1304	0.7183392	0.0052	0.9424765
Access to outside enclosure	1.4922	0.2230376	3.6912	0.0556340	2.6443	0.1050819
Minimal age	0.1005	0.7529559	**4.9321**	**0.0292637**	0.2916	0.5914110
Fecal cortisol (ng/g wet feces)	0.1355	0.71573	0.1129	0.7405706	0.8729	0.36057
VIM location x time period	**3.1999**	**0.0002561**	**3.0025**	**0.0003112**	**3.2476**	**0.0001627**

(B) Contrasts

Variable	Predictor	Level	Contrast	Estimate	SE	df	T.ratio	P-value
Shannon	VIM location and period	Post-hibernation period1	ex_situ - in_situ	0.312	0.0999	28.5	3.127	0.0111
		Post-hibernation period2	ex_situ - in_situ	0.302	0.1162	32.9	2.599	0.0360
		Pre-hibernation period1	ex_situ - in_situ	0.358	0.1125	37.6	3.185	0.0079
		Pre-hibernation period3	ex_situ - in_situ	0.363	0.1233	54.7	2.945	0.0129
		Summer	ex_situ - in_situ	0.441	0.1098	29.4	4.012	0.0011
			ex_situ - wild	0.559	0.1321	28.6	4.233	0.0006
	Previous location		birthplace - ex_situ	-0.287	0.092	22.6	-3.120	0.0130
	Birthplace		ex_situ - in_situ	0.4130	0.1296	22.89	3.187	0.0418
Faith's PD	VIM location and period	Summer	ex_situ - in_situ	4.737	1.48	35.0	3.195	0.0081
			ex_situ - wild	6.439	1.79	34.3	3.600	0.0028
Observed ASVs	VIM location and period	Post-hibernation period1	ex_situ - in_situ	30.8	10.33	29.1	2.982	0.0057
		Post-hibernation period2	ex_situ - in_situ	31.1	11.91	33.6	2.607	0.0135
		Post-hibernation period3	ex_situ - in_situ	29.1	12.82	58.8	2.270	0.0295
		Pre-hibernation period1	ex_situ - in_situ	39.9	11.56	38.1	3.449	0.0014
		Pre-hibernation period3	ex_situ - in_situ	34.0	12.60	54.0	2.698	0.0093
		Summer	ex_situ - in_situ	49.3	11.32	30.6	4.354	0.0004
			ex_situ - wild	61.4	13.63	29.9	4.501	0.0003
	Previous location		birthplace - ex_situ	-29.5	9.56	23.2	-3.086	0.0138
	Birthplace		ex_situ - in_situ	45.4	13.48	23.3	3.368	0.0278

(C) Generalized Additive Models

	**Pre-hibernation**				
Shannon	Predictor	Estimate	Std. Error	t-value	Pr(>|t|)
R2 adj=0.181	Intercept	4.53162	0.03031	149.495	< 2e-16
De=23.2%	Location: in situ	-0.15657	0.04991	-3.137	0.00216
	Smooth terms	Edf	Ref.df	F	p-value
	Days before hibernation:Location in situ	5.5220	6.6612	1.864	0.081

	**Post-hibernation**				
Shannon	Predictor	Estimate	Std. Error	t-value	Pr(>|t|)
R2 adj=0.253	Intercept	4.63500	0.02519	184.014	< 2e-16
De=27.2%	Location: in situ	-0.21932	0.03397	-6.457	2.59e-09
	Smooth terms	Edf	Ref.df	F	p-value
	Days before hibernation:Location in situ	0.6946	0.7219	1.224	0.349

	**Pre-hibernation**				
Faith's PD	Predictor	Estimate	Std. Error	t-value	Pr(>|t|)
R2 adj=0.065	Intercept	18.2939	0.4059	45.070	< 2e-16
De=8.76%	Location: in situ	-1.7212	0.6501	-2.648	0.00918
	Smooth terms	Edf	Ref.df	F	p-value
	Days before hibernation:Location ex situ	0.6667	0.6667	5.781	0.0519

	**Post-hibernation**				
Faith's PD	Predictor	Estimate	Std. Error	t-value	Pr(>|t|)
R2 adj=0.23	Intercept	19.2343	0.3506	54.865	< 2e-16
De=27.1%	Location: in situ	-2.8355	0.4989	-5.684	1.05e-07
	Smooth terms	Edf	Ref.df	F	p-value
	Days before hibernation:Location in situ	0.6667	0.6667	1.046	0.405

	**Pre-hibernation**				
Observed	Predictor	Estimate	Std. Error	t-value	Pr(>|t|)
R2 adj=0.163	Intercept	124.6	3.072	40.564	< 2e-16
De=20.9%	Location: in situ	-16.22	5.04	-3.219	0.00166
	Smooth terms	Edf	Ref.df	F	p-value
	Days before hibernation:Location in situ	4.5248	5.5524	1.830	0.109

	**Post-hibernation**				
Observed	Predictor	Estimate	Std. Error	t-value	Pr(>|t|)
R2 adj=0.257	Intercept	133.7	2.625	50.939	< 2e-16
De=28.2%	Location: in situ	-22.48	3.601	-6.241	7.49e-09
	Smooth terms	Edf	Ref.df	F	p-value
	Days before hibernation:Location in situ	1.6838	2.152	0.902	0.432

Table S3. Pairwise PERMANOVA models for significant variables in PERMANOVA models. Only values meeting the significance cutoff of p-value<0.05 are represented. Italicized rows show significant inter-individual variation through significant betadisper test.

Dataset	Variable	Level 1	Level 2	SumsOfSqs	MeanSqs	F.Model	R2	Pr(>F)
Summer	VIM Location	In situ	Wild	0.7519	0.75185	16.066	0.13608	1,00E-04
		In situ	Ex situ	0.6439	0.64390	14.111	0.14383	1,00E-04
		Ex situ	Wild	1.3961	1.39614	27.717	0.29575	1,00E-04
	Birthplace	*Ex situ*	*Wild*	*0.6462*	*0.64624*	*12.877*	*0.10924*	*1,00E-04*
		Ex situ	Ex & in situ	0.13516	0.135163	2.8837	0.05254	0.0021
		Ex situ	Ex situ & wild	0.12002	0.120024	2.565	0.05508	0.0092
		Ex situ	In situ	0.18131	0.181311	3.593	0.07245	7,00E-04
		Ex situ	In situ & wild	0.13721	0.137214	2.9468	0.06277	0.0016
		Wild	Ex & in situ	0.4363	0.43632	8.5083	0.10439	1,00E-04
		Wild	Ex situ & wild	0.2945	0.294497	5.6884	0.08047	2,00E-04
		Wild	In situ	0.3893	0.38932	7.1907	0.09692	1,00E-04
		Wild	In situ & wild	0.2390	0.239039	4.6311	0.06651	4,00E-04
	Parent's birthplace	Wild	Ex & in situ	0.3206	0.32062	5.8603	0.06906	1,00E-04
		Wild	In situ	0.1444	0.144447	2.5253	0.03632	0.0443
		Wild	In situ & wild	0.1297	0.129686	2.2729	0.03234	0.041
		Wild	Ex situ & wild	0.6622	0.66215	12.19	0.12942	1,00E-04
		Ex situ	Ex situ & wild	0.12582	0.125817	3.0404	0.13196	0.0072
		Ex situ & wild	In situ & wild	0.08194	0.081936	1.8844	0.08611	0.045
Hibernation	*VIM location & time period*	*Post-hibernation-ex situ*	*Post-hibernation-in situ*	*0.3341*	*0.33410*	*6.2236*	*0.0501*	*1,00E-04*
		Post-hibernation-ex situ	*Summer-ex situ*	*0.3885*	*0.38849*	*8.1309*	*0.10407*	*1,00E-04*
		Pre-hibernation-ex situ	*Summer-ex situ*	*0.5217*	*0.52172*	*8.8561*	*0.09144*	*1,00E-04*
		Post-hibernation-ex situ	*Pre-hibernation-ex situ*	*0.8393*	*0.83935*	*15.391*	*0.11042*	*1,00E-04*
		Post-hibernation-in situ	*Pre-hibernation-in situ*	*0.3178*	*0.31778*	*4.5551*	*0.03942*	*1,00E-04*
		Pre-hibernation-ex situ	*Pre-hibernation-in situ*	*0.6089*	*0.60895*	*8.719*	*0.06935*	*1,00E-04*
		Summer-in situ	*Summer-ex situ*	*0.5494*	*0.54943*	*11.561*	*0.12627*	*1,00E-04*
		Summer-in situ	*Pre-hibernation-in situ*	*0.5421*	*0.54208*	*8.703*	*0.07394*	*1,00E-04*
		Summer-in situ	*Post-hibernation-in situ*	*0.7582*	*0.75822*	*14.289*	*0.10042*	*1,00E-04*
	Birthplace	Ex situ	Wild	0.4951	0.49511	8.1246	0.03942	1,00E-04
		Ex situ	Ex & in situ	0.4252	0.42519	6.9039	0.0356	1,00E-04
		Ex situ	Ex situ & wild	0.3658	0.36582	6.1202	0.03752	1,00E-04
		Ex situ	In situ	0.3752	0.3752	6.0224	0.03649	1,00E-04
		Ex situ	In situ & wild	0.2834	0.283372	4.6317	0.02848	1,00E-04
		Wild	Ex & in situ	0.5899	0.58987	9.5236	0.07773	1,00E-04
		Wild	Ex situ & wild	0.5715	0.57149	9.7472	0.10509	1,00E-04
		Wild	In situ	0.4855	0.48546	7.6583	0.08265	1,00E-04
		Wild	In situ & wild	0.3830	0.38299	6.2483	0.06923	1,00E-04
		Ex situ & wild	In situ & wild	0.18822	0.188221	3.2805	0.07088	1,00E-04
		In situ	In situ & wild	0.3013	0.30125	4.5356	0.09156	2,00E-04
	Parents' birthplace	Wild	Ex & in situ	0.2537	0.253671	3.911	0.03403	4,00E-04
		Wild	In situ & wild	0.4390	0.43896	6.5602	0.05677	1,00E-04
		Wild	Ex situ & wild	0.464	0.464	7.1384	0.04661	1,00E-04
		Ex & in situ	In situ & wild	0.3074	0.307364	4.9397	0.0642	1,00E-04
		Ex & in situ	Ex situ & wild	0.3916	0.39164	6.3939	0.05541	1,00E-04
		In situ & wild	Ex situ & wild	0.1494	0.149357	2.3604	0.02158	0.0066

Table S4. Results of differential abundance analysis from both ANCOM-BC and MaAsLin2 meeting the significance cutoff of p-value<0.05

Supplementary document. V. I. Marmot fecal cortisol metabolite concentration determination through enzyme immunoassay.

V. I. Marmot fecal cortisol metabolite concentration determination through enzyme immunoassay

Methods:

Hormone extraction and analysis:

A total of 34 VIMs were sampled for fecal cortisol (N_{wild} = 15, $N_{in\ situ}$ = 15, $N_{ex\ situ}$= 4) at the Toronto Zoo. Hormones were extracted from 0.2-0.5 g of feces by adding 0.1 g/ml of 80% methanol-distilled water overnight (16-18 h) at room temperature with continuous mixing on a plate shaker. Samples were centrifuged for 10 min at 2,400 rcf and the supernatant (fecal extract) was decanted and stored in tightly capped glass vials at −20°C until analysis.

Prior to hormone quantification, a portion of the fecal extracts were evaporated to dryness in a fume hood at room temperature, then reconstituted with EIA buffer (0.1 M sodium phosphate buffer, pH 7.0, containing 9 g/L of NaCl and 1 g/L bovine serum albumin) for one hour with vortexing (5 sec) at the beginning and end of reconstitution. Evaporated extracts were reconstituted in EIA buffer at 1:3-1:10 for fecal cortisol metabolite (FCM) analysis.

FCM concentration was determined by enzyme immunoassay following the method previously described (Majchrzak et al. 2015) with modifications. Cortisol-horseradish peroxidase (HRP) and polyclonal antibody (R4866; C. Munro, University of California, Davis, CA, USA) were diluted 1:33,500 and 1:10,000 in EIA buffer, respectively. Briefly, microtitre plates (Nunc Maxisorp, VWR, Mississauga, ON, Canada) were coated with 50 µl of cortisol antibody diluted in coating buffer (50 mM bicarbonate buffer, pH 9.6) and incubated overnight at 4 °C. Unbound antibody was washed from coated plates with 0.05% Tween 20, 0.15 M NaCl solution using a Bio-Tek ELx 405VR microplate washer (Bio-Tek Instruments, Winooski, VT) and 50 µl of reconstituted extracts, standards, and controls diluted in EIA buffer were added in duplicate followed by 50 µl of horseradish peroxidase conjugate. Plates were incubated for 2 h at room temperature, then washed and 100 µl of substrate solution (125 µl of 40 mM 2,2'-azino-bis(3-ethylbenzothiazoline-6-sulfonic acid) diammonium salt and 40 µl of 0.5 M H2O2 diluted in 12.5 ml 50 mM citrate buffer, pH 4.0) was added. Absorbance was measured at 405 nm using a spectrophotometer (BioTek EPOCH 2 microplate reader and Gen5 3.08 software, Agilent Technologies, Santa Clara, CA, USA). The cross-reactivities of the cortisol antibody were previously reported (Young et al. 2001). All samples and standards were run in duplicate. Only values from duplicates with $<10\%$ CV were recorded as data. Data are presented as nanograms of hormone per gram of feces (ng/g).

Enzyme immunoassay validation

Parallel displacement between the standard curve and serial dilutions of fecal extract was used as an indirect measure of assay specificity. Pooled reconstituted fecal extracts were serially diluted two-fold in assay buffer and visually compared to the respective standard curve. The data were plotted as log(relative dose) vs. percent antibody bound (Microsoft Excel). The slopes of the lines within the linear portion of the curves were determined using linear regression analysis and compared (Soper 2021) where $p>0.05$ indicates the slopes are not significantly different and thus interpreted as parallel. Samples were assayed at the dilution factor that corresponded to 50% binding of the serially diluted fecal pool.

Recovery of known amounts of cortisol was calculated to examine possible interference of fecal extract components with antibody binding. Increasing concentrations of hormone standard were added to an equal volume of pooled fecal extracts of known concentration. The percent recovery was calculated using the formula: amount observed/amount expected x 100%, where amount observed is the value from the spiked sample minus the endogenous hormone in the unspiked extract, and the amount expected is the concentration of hormone standard added. The percent recovery is presented as mean ± SE.

Results:

Serial dilutions of pooled fecal extract showed parallel displacement with the cortisol standard curve ($t=1.70$, $p=0.13$, $df=7$; Fig 1). The recovery of known concentrations of cortisol was 103.6% ± 4.2 (mean ± SE). The measured hormone concentrations in the spiked samples correlated with the expected concentrations of cortisol ($r=0.99$, $p<0.001$; Fig 2). Inter-assay CVs were 13.2% and 10.3% (25% and 62% binding, respectively).

Majchrzak, Y.N., Mastromonaco, G.F., Korver, W., Burness, G., 2015. Use of salivary cortisol to evaluate the influence of rides in dromedary camels. Gen. Comp. Endocrinol. 211, 123–130. doi:10.1016/j.ygcen.2014.11.007

Soper, D.S. 2021. Significance of the Difference between Two Slopes Calculator [Software]. Available from https://www.danielsoper.com/statcalc

Young, K.M., Brown, J.L., Goodrowe, K.L., 2001. Characterization of Reproductive Cycles and Adrenal Activity in the Black-footed Ferret (Mustela nigripes) by Fecal Hormone Analysis. Zoo Biology 20: 517–536. doi:10.1002/zoo.10001

Figure 1: Fecal cortisol parallelism test for the Vancouver Island Marmot.

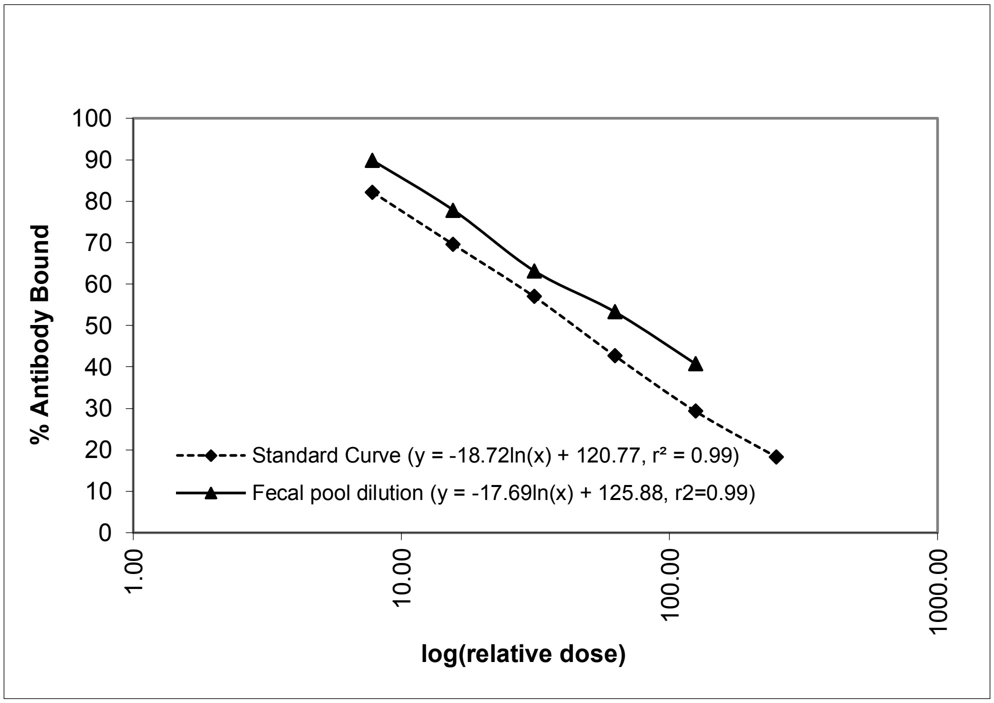

Fig 2: Fecal cortisol recovery and accuracy test for the Vancouver Island Marmot.

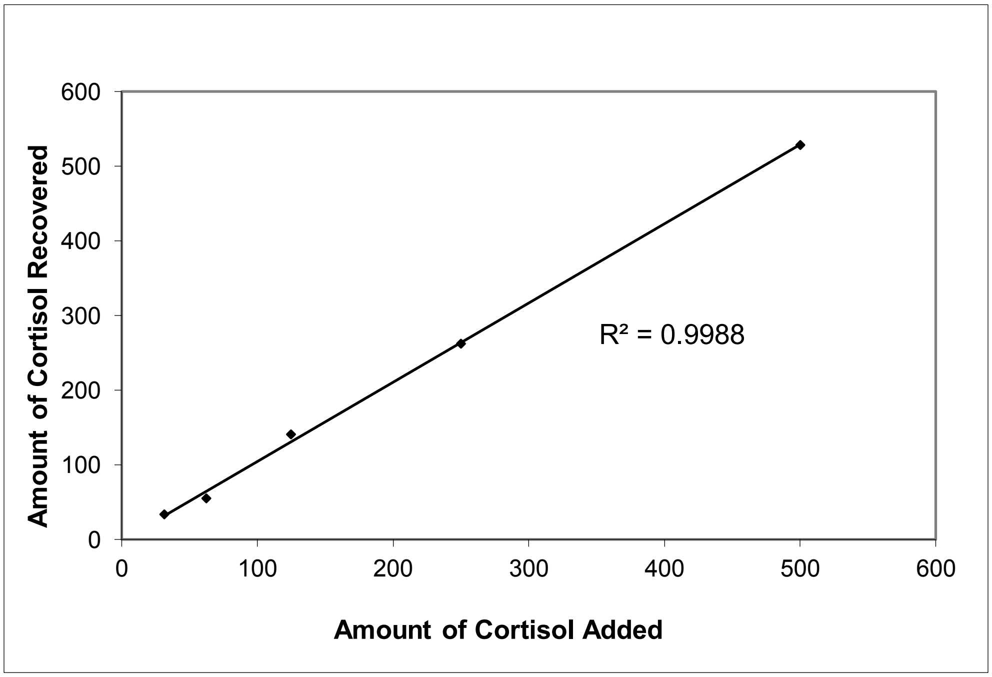

Supplement: Web_Material_coae072 [file web_material_coae072.zip › VIM-SUPPMAT.pdf]
